# Genetic Structure and Expression of the Surface Glycoprotein GP82, the Main Adhesin of *Trypanosoma cruzi* Metacyclic Trypomastigotes

**DOI:** 10.1155/2013/156734

**Published:** 2013-02-04

**Authors:** Paulo Roberto Ceridorio Correa, Esteban Mauricio Cordero, Luciana Girotto Gentil, Ethel Bayer-Santos, José Franco da Silveira

**Affiliations:** Departamento de Microbiologia, Imunologia e Parasitologia, Escola Paulista de Medicina, Universidade Federal de São Paulo, Rua Botucatu 862, 04023-062 São Paulo, SP, Brazil

## Abstract

*T. cruzi* improves the likelihood of invading or adapting to the host through its capacity to present a large repertoire of surface molecules. The metacyclic stage-specific surface glycoprotein GP82 has been implicated in host cell invasion. GP82 is encoded by multiple genes from the trans-sialidase superfamily. GP82 shows a modular organization, with some variation of N-terminal region flanking a conserved central core where the binding sites to the mammalian cell and gastric mucin are located. The function of GP82 as adhesin in host cell invasion process could expose the protein to an intense conservative and selective pressure. GP82 is a GPI-anchored surface protein, synthesized as a 70 kDa precursor devoid of *N*-linked sugars. GPI-minus variants accumulate in the ER indicating that GPI anchor acts as a forward transport signal for progressing along the secretory pathway as suggested for *T. cruzi* mucins. It has been demonstrated that the expression of GP82 is constitutive and may be regulated at post-transcriptional level, for instance, at translational level and/or mRNA stabilization. GP82 mRNAs are mobilized to polysomes and consequently translated, but only in metacyclic trypomastigotes. Analysis of transgenic parasites indicates that the mechanism regulating GP82 expression involves multiple elements in the 3′UTR.

## 1. Introduction

*Trypanosoma cruzi* is a protozoan parasite that causes Chagas disease, also called American trypanosomiasis, a debilitating and incurable disease affecting millions of people in Latin America. The life cycle of *T. cruzi* has multiple developmental stages: two in the invertebrate vector (triatomine hematophagous insects) and two in the vertebrate hosts. The infective forms are the trypomastigote stages found in the bloodstream of mammalian hosts and the metacyclic trypomastigotes present in the digestive tract of triatomines. Metacyclic trypomastigotes when eliminated in the feces of the triatomine can initiate the infection of mammalian hosts by invading a variety of cell types. They express a stage-specific surface glycoprotein of 82 kDa (GP82) involved in host cell invasion that has no counterpart in bloodstream trypomastigotes [1–4]. GP82 is the major cell adhesion molecule of metacyclic forms that induces the activation of Ca2^+^ signaling cascades, leading to host cell cytoskeleton rearrangement and recruitment of lysosomes at the site of parasite entry, events required for the formation of the parasitophorous vacuole, and parasite internalization [1, 2, 5–8]. In this paper, we will mainly focus on the genetic structure of GP82 family and regulation of its expression by post-transcriptional control mechanisms.

## 2. Structure of *GP82* Gene

**Figure 1:**
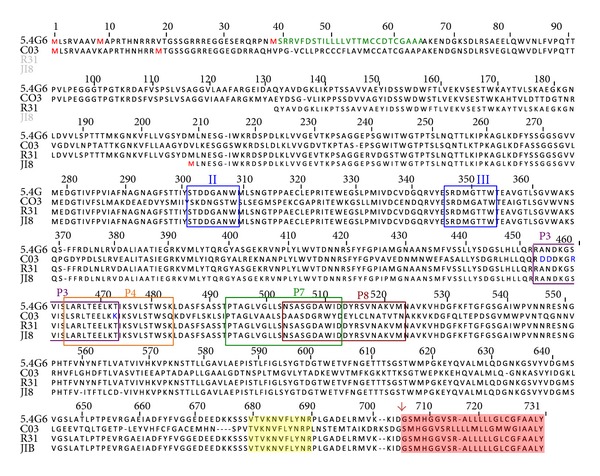
Alignment of amino acid sequences of some representatives of the GP82 family. Sequences are encoded by cDNA clones isolated from *T. cruzi* metacyclic trypomastigotes: 5.4G6 (ABR19835); CO3 (ABO28970); R31 (AF128843); J18 (AAA21303). GenBank accession numbers are in parentheses. Potential initiator methionines (M) and a predicted N-amino terminal signal peptide are indicated in red and green, respectively. The Asp boxes (bacterial sialidase motifs) are boxed and indicated by Roman numerals II and III. The epitope for Mab3F6 (P3), mammalian cell binding sites (P4 and P8), and gastric-mucin binding site (P7) are boxed and indicated by different colors. Note the overlapping between P3 and P4 sites, and P7 and P8 sites. The subterminal VTV motif, characteristic of the TS superfamily, and the potential GPI-anchor sequence are shaded in yellow and magenta, respectively. The arrow denotes the cleavage site for GPI anchor addition.

The GP82 glycoprotein was first identified in the cellular surface of metacyclic forms by the monoclonal antibody Mab3F6 generated by immunization of mice with intact, heat inactivated *T. cruzi *metacyclic forms [3, 4]. Since the determination of the first *GP82* gene sequence in 1994 [9] many other sequences have become available [10–14], including those from *T. cruzi* genome sequencing projects [15–17]. The original analysis by Araya et al., 1994, showed the presence of two highly conserved Asp box domains (SxDxGxTW), previously described in bacterial sialidases, and a subterminal (VTVxNVFLYNR) motif (Figure 1) that are characteristics of the trans-sialidase (TS) superfamily of *T. cruzi* [18]. For this reason GP82 was classified in the TS superfamily [9, 18].

**Figure 2:**
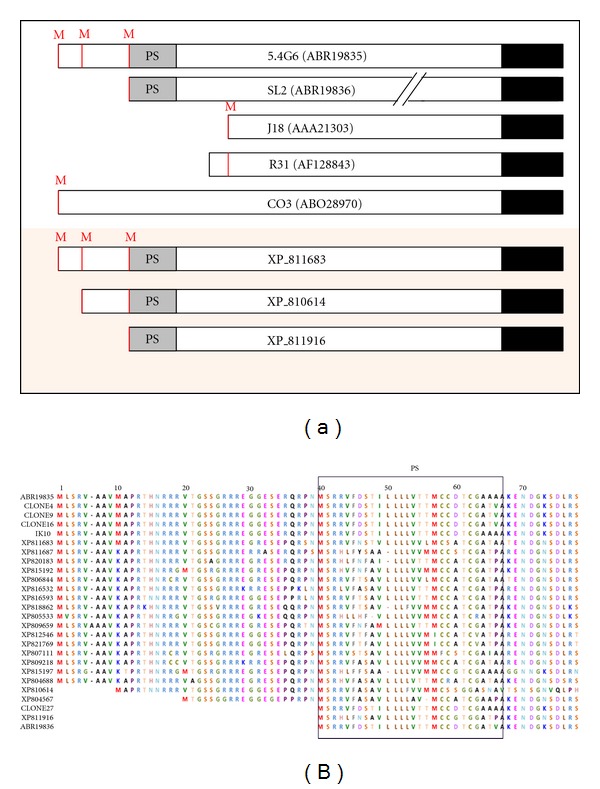
The modular architecture of GP82 family. (a) Structure of GP82 core proteins deduced from cDNA and genomic sequences. Sequences from cDNA clones are listed in the legend of Figure 1. SL (ABR19836) is a truncated cDNA sequence obtained by RT-PCR. The slashes indicate that sequence is interrupted. For ease of viewing, the putative C-terminal was drawn in the same line. GenBank accession numbers are in parentheses. The genomic sequences (GeneBank: XP_811683, XP_810614, XP_811916) were from the *T. cruzi *genome sequencing project (clone CL Brener). Potential initiator methionines (M), predicted N-amino terminal signal peptide (SP), and potential GPI-anchor sequence are indicated in red, gray, and black, respectively. Not drawn to scale. (b) Alignment of GP82 sequences showing the variation of N-terminal region. Potential initiator methionines (M) are indicated in red and the predicted N-amino terminal signal peptide (PS) is boxed. cDNA sequences: ABR19835, clones 4, 9, 16, IK10 and 27, ABR19835, ABR19836; genomic sequences were extracted from the *T. cruzi *genome sequencing project (clone CL Brener) and are indicated by the prefix XP_.

Figure 2 shows the comparison of five GP82 sequence variants isolated in our laboratory by cDNA cloning and three genomic sequences of clone CL Brener (*T. cruzi* genome project). Although all variants code for a glycosylphosphatidylinositol (GPI) anchor addition signal sequence at the carboxy-terminal (C-terminal), several of them do not have a signal peptide sequence at the amino-terminal (N-terminal), suggesting that they are not translocated into the endoplasmic reticulum (ER) and do not receive the GPI anchor.

Among the sequences annotated as GP82, the cDNA clone 5.4G6 (GenBank: EF154827) represents a complete transcript including the 5′ and 3′ untranslated regions (UTRs) (Figures 1 and 2) [12]. This clone encodes a protein of 726 amino acids (GenBank: ABR19835) that reacts with the Mab3F6 (Figure 1) [12]. The open reading frame (ORF) has three ATG initiation codons in the same reading frame, but only the third codon is inserted within the Kozak sequence context (Figure 2) [12]. It has been proposed that the Kozak sequence (consensus: GCCRCCaugG, R designates a purine base and aug the initiation codon), located upstream of the initiation codon, facilitates the mRNA translation [19]. Furthermore, clone 5.4G6 encodes an N-terminal signal peptide of 27 amino acids located just after the third methionine at position 39 and a signal sequence for cleavage/addition of GPI anchor at the C-terminal (Figures 1 and 2). Taking into account that the third ATG initiation codon follows the consensus Kozak sequence and the encoded protein has a predicted signal peptide, it is presumed that the translation initiates at the third methionine (Figure 2). The presence of 2-3 initiation codons in the same reading frame is relatively common among *T. cruzi* surface proteins, and after the second or the third methionine there is a sequence encoding a cryptic signal peptide as described in many genes as GP85 [20], Tc85 [21], ASP-2 [22], CRP-10 [23], GP90 [24], and SAP [25].

The existence of GP82 sequences lacking a typical N-terminal signal peptide suggests that its products may be located intracellularly, as occurs with the GP82 encoded by the clone C03 (Figure 1) which is located at the flagellum of metacyclic forms of the CL strain [13]. Such GP82 protein displaying flagellar localization is not involved in the invasion of mammalian cells by metacyclic forms [13]. Data obtained with monospecific anti-GP82 antibodies support the hypothesis that GP82 proteins that have no N-terminal signal peptide are located intracellularly and are not involved in host cell invasion [13].

The members of TS superfamily show a highly conserved hydrophobic sequence (M S R R V F/T S V L L L L F/L V) at the N-terminal region, which acts as a signal peptide addressing the nascent protein into the ER. Analysis of the N-terminal region of the CRP-10 from TS superfamily indicates that this sequence functions as signal peptide [23]. Based on the analysis of GP82 variants characterized to date, we could suggest that metacyclic trypomastigotes simultaneously express different variants of GP82, and their cellular localization is determined by the N-terminal signal peptide. However, as the N-terminal of native GP82 has not been determined, we cannot rule out the possibility that the translation starts in different ATG codons.

The epitope recognized by Mab3F6 and the site of adhesion of GP82 to the host cells were identified by incubating recombinant proteins and synthetic peptides in mammalian *in vitro* invasion assays. Regions of GP82 gene coding for the C-terminal, central, and N-terminal domains were subcloned into plasmid pGEX and expressed in *E. coli*. The reactivity with Mab3F6 and the ability of each recombinant protein to inhibit cell invasion were tested [6], both the Mab3F6 (P3) binding site and the host cell adhesion sites (P4 and P8) were identified in the central domain of GP82 (Figure 1) as will be discussed in the topic below.

## 3. Organization of *GP82* Gene Family

*T. cruzi* genome comprises more than 50% of repetitive sequences including several multigene families that encode surface proteins. Among them, the most abundant is the TS superfamily [17]. The genome sequencing of clone CL Brener [17] confirmed the complexity of the TS superfamily by identification of 1,430 sequences, including 737 genes and 693 pseudogenes. These sequences have been annotated as trans-sialidase in the *T. cruzi *genome project with no mention to which group or family they could be included.

According to sequence identity, molecular weight, and function, members of TS superfamily were classified into four groups or families [18, 26–29]. Members of a family or group exhibit ≥60% similarity among each other, whereas similarity among members of different families or groups may vary from 20 to 40%. Group I of the TS superfamily comprises proteins with enzymatic activity; that is, they are enzymes (trans-sialidase) able to transfer sialic acid from a donor to the mucins present at *T. cruzi* surface [18, 26–29].

The members of group II were also called “trans-sialidase like” proteins because they have no enzymatic activity [18, 26–29]. These proteins have complete or degenerate Asp box motifs (SxDxGxTW), the VTVxNVFLYNR motif characteristic of all TS members, and the signal sequence for cleavage/addition of GPI anchor at the C-terminal region [18, 26–29]. Group II comprises the surface glycoproteins GP85, Tc85, TSA-1, SA85, GP90, GP82, ASP-1, and ASP-2 which are involved in adhesion and invasion of mammalian cells [18, 20–22, 24, 26–30]. Several proteins of this group are also targets of the host immune system and may induce protective immunity in animal models [30]. The proteins of group III (CRP, FL160, CEA, and TESA) inhibit the classical and alternative pathways of complement activation and are recognized by sera from patients with Chagas disease [18, 26–29, 31, 32]. Group IV is composed of genes encoding trypomastigote surface antigens that have no defined biological function [18, 26, 27].

Recently, Freitas et al., 2011, [33] reported an extensive and detailed analysis of TS sequences of clone CL Brener that resulted in the redistribution of members in 8 different groups designated as TcSgroupI to TcSgroupVIII. The sequences analyzed in this study (*n* = 508) were categorized according to structure, function, presence of conserved motifs, chromosomal localization, expression profiling, and antigenic properties. TcSgroupI to TcSgroupIV (*n* = 176) correspond to groups I to IV described above. There is a good correlation with the classification proposed previously [18, 26, 27] and with the prior annotation made in our laboratory [16]. The new classification proposed by Freitas et al., 2011, [33] could categorize 329 sequences that were included in the groups TcSgroupV–TcSgroupVIII.

To identify the repertoire of *GP82* genes in the genome of clone CL Brener (the reference clone of *T. cruzi* Genome Project), we carried out a BLASTP search using the GP82 encoded by clone 5.4G6 (GenBank: ABR19835) as query [10]. We identified 19 complete sequences with >60% identity with the query which were considered as GP82 and distributed as follows: 2 proteins (GenBank: XP_811663 and XP_804688) with 70–81% identity and the remaining with 61–68% identity (see Figure 2). Pseudogenes and truncated sequences were discarded from the analysis. Although GP82 are encoded from a relatively small number of genes, the repertoire is quite variable. This contrasts with other TS-like protein families which are composed of large sets of genes such as GP85, Tc85, GP90, and ASP [16, 20, 21, 24, 33].

The ability of genes to be robust to mutations at the codon level has been suggested as a key factor for understanding adaptation features. It has been proposed that genes relevant to host-parasite interactions will tend to exhibit high volatility or “anti-robust” patterns, which may be related to the parasite capacity of evading the host immune system [34]. We investigated the potential capacity of *T. cruzi *surface protein genes to maximize phenotypic variation, which may be seen as a key attribute to expand the repertoire of surface antigens [34]. The robustness of a parasite gene against mutations was addressed in terms of several gene *volatility *and *diversity *indicators. The potential impact of point-mutation errors on surface antigen genes based on the analysis of codon usage and its potential for generating different amino acid mutants were explored. These data were consistent with the low rate of volatility calculated using the GP82 sequences deposited in GenBank [16]. *GP82* genes have “low volatility” which means that the mutations are generally synonymous or lead replacing amino acids with others of the same polarity. *GP82* genes seem to be genetically “robust”; that is, they exhibit a tendency to neutralize the mutations encoding the same amino acid or an amino acid of the same polarity.

Analysis of selective pressure on GP82 variants showed that the protein could have undergone conservative or negative selection. The function of GP82 as adhesin in host cell invasion process could expose the protein to an intense conservative and selective pressure. The potential variability of *GP82* genes suggests that they are “robust” or are not susceptible to mutation [16].

In the human protozoan parasites *Trypanosoma brucei* and *Plasmodium falciparum,* the subtelomeric regions play an important role in generation of new variants of surface antigen genes and in the control of gene expression [35–37]. We reported that *T. cruzi* subtelomeric regions are enriched in (pseudo)genes from the TS superfamily, DGF-1, and retrotransposon hot spot protein (RHS) families [38, 39]. The abundance of surface protein genes in the subtelomeric regions suggests that these regions may have acted as a site for DNA recombination, expansion, and the generation of new variants of surface proteins. Moraes Barros et al. (2012) [39] demonstrated that all the groups of the TS superfamily are represented in the subtelomeric regions of clone CL Brener; most of the sequences (*n* = 83) are members of group II (GP82, GP85, TC85), which includes 22 complete genes. It is interesting to note that 7 out of 19 *GP82* genes identified in clone CL Brener are located at subtelomeric regions.

## 4. Synthesis and Processing of GP82

GP82 is attached to the outer parasite's cell membrane by a GPI anchor [3, 40]. It is synthesized as a 70 kDa precursor devoid of *N*-linked sugars and when mature, it has an apparent molecular weight of 82 kDa. GP82 binds to the target cell in a dose-dependent and saturable fashion and reduces the infection of Vero cells by metacyclic forms of CL and Tulahuen strains by 90 to 97 and 50%, respectively [8].

The immunological screening of a metacyclic cDNA library with the Mab3F6 allowed the isolation of a 2,140 bp clone, named J18 (GenBank L14824), which encodes a protein of 516 amino acids containing the functional domains of GP82 [9]. Analysis of the deduced amino acid sequence showed the presence of three sialidase domains (two conserved and one slightly degenerated), a VTV motif, four putative *N*-glycosylation sites, and a GPI-anchor addition signal, which allows us to classify the GP82 in group II of TS superfamily [9]. 

Recombinant expression of clone J18 and a series of step-wise deletions enabled the identification of the domain involved in the adhesion to the mammalian cells and indirectly, the region containing the epitope for Mab3F6 [6]. A central region spanning 132 amino acids was identified as the responsible for the adhesin properties and ten overlapping peptides encompassing this central domain were synthesized to further characterize the region. The authors found two non-contiguous peptides with significant adhesive properties, named P4 and P8; thus, they speculated about a putative conformational binding-domain in the native protein, in which these two peptides would be in close proximity [6].

To further address the adhesin activity of these peptides (P4 and P8) and to rule out any peptide's solubility and conformational artifacts, both peptides were expressed in a non-adherent microorganism. The expression of GP82 cell binding peptides P4 or P8 in the fourth surface-exposed loop of the transmembrane protein LamB of *Escherichia coli* conferred the ability to this microorganism to adhere to the surface of HeLa cells [41]. Between the two populations of bacteria, those carrying the P4 peptide were almost twice more efficient to adhere to HeLa cells than the population expressing the P8. In the same way, the expression of GP82 protein on the outer membrane of non-infective *T. cruzi* epimastigotes enabled these non-adherent forms to attach to the surface of HeLa cells [42]. 

A more detailed analysis on the central domain of GP82 was performed by Manque et al., 2000, [43] using the same peptides described above by Santori et al., 1996, [6] and variants of GP82 lacking the regions corresponding to the peptides P4 and P8. This strategy allowed the authors to identify the peptide P3 as the epitope recognized by Mab3F6. As the peptide P3 has ten amino acids overlapping with the peptide P4 (cell binding site), this finding provided support for the inhibition of parasite's invasion by Mab3F6, which is probably due to sterical hindrance. Additionally, the authors were able to confirm the GP82 conformational cell-binding domain hypothesis raised by Santori et al., 1996, [6] by means of the hybrid peptide P4/8 which contained 17 amino acids from P4 and 5 amino acids from P8 peptide. This peptide P4/8 was more efficient than P4 and P8 peptides to inhibit the binding of the recombinant GP82 to the HeLa cells [43].

Recently, it was demonstrated that GP82 binds specifically to gastric mucin in the oral infection [44, 45]. The implication of GP82 in adhesion of metacyclic forms to the gastric mucin was first described by Neira et al., 2003, [45] and further confirmed by Staquicini et al., 2010, [44] using a GP82 recombinant protein Del-4/8 lacking the central domain of the molecule. The GP82 binding to the gastric mucin may direct the adhesion and invasion of the stomach epithelium by the metacyclic forms. Tests of invasion inhibition of the gastric mucosa showed that peptide P7 (Figure 1), located in the central domain of the molecule, contains the binding site to the gastric mucin [44]. The *in vitro* inhibitory effect of peptide P7 was reproducible *in vivo* in murine model [44].

Experimental evidence of the GP82 GPI-anchor was given by Cardoso De Almeida & Heise (1993) [40] through digestion with phosphatidylinositol-specific phospholipase C (PI-PLC) and phase separation in Triton X-114. Araya et al., 1994, [9] predicted the putative GP82 GPI-anchor cleavage/addition site based on the sequence of the clone J18. Similarly, Ramirez et al., 1999, [14, 46] analyzed in more detail the GPI-anchor signal of GP82 and other *T. cruzi* proteins and conducting homologous and heterologous expressions of GP82 in *T. cruzi* epimastigotes and mammalian cell systems. Despite the absence of a typical signal peptide in the protein encoded by the J18 clone, the authors found that *T. cruzi* machinery was able to translocate the protein inside the ER finally deliver it to the parasite's cell surface (Ramirez et al., 1999) [14]. On the other hand, when the same protein was synthesized by mammalian cells, it failed to translocate inside the ER and accumulated in the cytoplasm, indicating the requirement for a typical signal peptide. When the required signal was provided by insertion of the signal peptide from the influenza virus hemagglutinin, the mammalian cell was able to translocate the GP82 chimera inside the ER but proved insufficient to provide expression on the cell surface. These findings indicated that the requirements for GPI-anchoring are different between *T. cruzi* and mammalian cells [14, 46].

In order to further dissect the requirements for GPI-anchoring between mammals and *T. cruzi*, a site-directed mutagenesis was performed in the GPI cleavage/addition signal [47]. The putative GPI-anchor acceptor domain determined by Ramirez et al., 1999, [46] is formed by the amino acids aspartic (*ω*), glycine (*ω* + 1), and serine (*ω* + 2) (DGS) where the aspartic acid is linked to the GPI-anchor. A single mutation was introduced changing the aspartic acid to serine generating the sequence (SGS) which previously proved to be a feasible signal for GPI anchoring of *T. brucei* Variant Surface Glycoprotein (VSG) in mammals [48]. An additional construct lacking the GPI-anchor was created and transfected either in mammalian cells or *T. cruzi* epimastigotes [47]. 

Confocal analyses on transfected parasites showed that the point mutation had no detectable effect on the GPI-anchoring efficiency [47]. The deletion of the GPI-signal resulted in a protein that was not anchored but accumulated in the parasite cytoplasm instead [47]. These findings were in agreement with those obtained in *T. brucei* by Böhme and Cross (2002) [49] where the parasite was able to anchor several mutated proteins but not those in which the GPI-anchor signal was deleted. On the other hand, the mammalian cells failed to express all the transfected proteins on the cell surface, even the point mutation which proved to be functional for GPI anchoring in mammals [48]. 

Isolation of GPI-anchored proteins can be accomplished by digestion with enzymes that cleave specifically this structure as glycosylphosphatidylinositol-specific phospholipase C (GPI-PLC) or phosphatidylinositol-specific phospholipase C (PI-PLC). This very simple approach can be hindered by the presence of acylation in position 6 of the inositol ring (sometimes the acylation occurs at position 5) due to steric hindrance.

Due to the presence of the GPI anchor, proteins carrying this modification acquire an overall hydrophobic behavior. Based on this property, detergents can be used to concentrate/enrich fractions in this particular kind of proteins by temperature-induced partition. Bordier 1981 [50] first described the suitability of the detergent Triton X-114 (TX-114) to concentrate hydrophobic proteins due to its nearly physiological clouding point temperature. At temperatures above 23°C a formerly homogenous solution containing the detergent TX-114 will split into two phases: an upper layer depleted of detergent (hydrophilic) and a lower phase enriched in detergent's micelles (hydrophobic). Using this physical property, Cordero et al., 2009, [51] concentrated the GPI-anchored proteins of metacyclic and epimastigotes of *T. cruzi* after several consecutive partitions in TX-114. Mass spectrometry analyses on those fractions detected several members of TS superfamily, among those, the surface glycoprotein GP82 had 22% of its sequence covered by tryptic peptides. Among those peptides, the Asp boxes, VTV, and cell binding site P8 were mapped [51]. Other important region mapped in this study was peptide P7, which was identified as the binding site for the gastric mucin [44]. 

Several proteomics studies have been conducted in metacyclic forms of *T. cruzi*, but to the best of our knowledge, there is only one additional report of GP82 elsewhere [52]. Recently, a quantitative proteomic study was performed in parasites undergoing metacyclogenesis [53]. Among the proteins identified in this study, authors found 38 members of TS superfamily. Due to lack of a unified/standardized annotation among the databases and the absence of the peptide sequences used in this study, it was not possible to determine the presence of GP82 among them. Some of those annotated TSs shared a high degree of identity with GP82 protein, but because of the missing peptide sequences it is impossible to assign an unambiguous classification. The lack of a common nonredundant annotation represents an issue that must be taken in consideration with an urge to amend.

Recently, Cortez et al., 2012, [54] compiled all the biochemical, physicochemical, and functional information available on GP82 in order to create the most updated model of the protein structure. The authors based this model on the homology of *T. cruzi* GP82 (GenBank: L14824) with *T. rangeli* sialidase (PDB 1N1T_A), a close related molecule which had its crystallographic structure (inhibitor-bound) already solved. The sketched GP82 appears as two clearly different and separated domains (an amino-terminal *β*-propeller and a *β*-sandwich C-terminal domain) linked together by an *α*-helix. In this layout, P3, P4, P7, and P8 motifs have a variable degree of access to the solvent. The cell-binding peptide P4 encompasses 2/3 of the *α*-helix that bridges the protein together and is fully exposed. On the other hand, peptide P8 located in the carboxy-terminal domain, although exposed, has limited solvent accessibility. The partial exposure of the P8 motif complies with the experimental data and gives a topological explanation for the limited role of P8 in the GP82 binding to the cell [41]. As expected, the P3 motif containing the epitope for the 3F6 antibody was fully exposed and accessible to the solvent, reinforcing the Mab3F6 inhibitory effect by steric hindrance. The gastric mucin-binding motif P7 was poorly exposed, mostly due to its high hydrophobicity. The amino-terminal residues of this motif (P7) are completely buried, leaving just the C-terminal portion partially exposed, mainly because it overlaps with the P8 motif. In summary, this model seems to fulfill the requirements for structural analysis and provides an appropriated support to the biological experimental data.

## 5. Post-Transcriptional Control Mechanisms of GP82 Expression

In trypanosomes, post-translational control mechanisms play an important role in gene expression regulation due to unique features related to transcription, mRNA maturation, and stabilization over the parasite life cycle. *T. cruzi* genome is organized in large gene clusters separated by divergent strand-switch regions [55], and transcription of these clusters produces large primary transcripts that are processed by trans-splicing and polyadenylation to generate mature mRNAs [56]. These processes are guided by pyrimidine-rich regions contained in the polycistronic transcripts [57, 58]. Although there is no transcription regulation in *T. cruzi*, proteomic analysis of the four developmental stages (epimastigote, metacyclic trypomastigote, amastigote, and bloodstream trypomastigote) demonstrated that there was a significant change in relative protein abundance throughout life cycle [52]. Furthermore, microarray analysis showed that at least 50% of *T. cruzi* genes are regulated during its life cycle [59]. GP82 is one of these differentially regulated proteins, and the mechanisms regulating its stage-specific expression began to be clarified.

Steady-state levels of GP82 transcripts from *T. cruzi* G strain were determined by northern blot, dot-blot hybridization, and quantitative real-time PCR, demonstrating that there is a significant increase in GP82 mRNA levels in metacyclic forms when compared with the other three stages [9, 11, 60]. Northern blot analysis revealed a single band of 2.2 kb mRNA only in metacyclic forms [9]. Dot-blot hybridization showed that GP82 transcript levels were around 5.5-fold higher in metacyclic trypomastigotes than in other stages [60]. Similar results were obtained using quantitative real-time PCR (unpublished data). Moreover, expression analysis of other three GP82 gene subfamilies from Peru-2 strain, called groups A, B, and C, showed an increase in mRNA accumulation (4.7 to 9.3-fold) in metacyclic forms when compared to epimastigotes [11]. Additionally, GP82 protein stage-specific expression was also showed by western blot using the Mab3F6 [3]. Even though GP82 mRNA and protein were barely detected in epimastigotes, nuclear run-on analysis demonstrated that *GP82* gene was transcribed in both epimastigote and metacyclic forms, confirming that transcript accumulation in metacyclic forms is not due to an increased transcription rate, but rather to some post-translational control [60]. 

Changes in GP82 mRNA stability were detected and thought to be responsible for differences in its steady-state level. Parasites treated with actinomycin D had their GP82 transcript half-lives estimated to be about 6 h in metacyclic forms and 0.5 h in epimastigotes [60]. Cycloheximide treatment increased GP82 levels in epimastigotes, suggesting that a labile protein factor was responsible for destabilizing mRNA in these forms and prevent mRNA translation. In addition, GP82 mRNAs were only found associated with polysomes in metacyclic forms [60], indicating that transcript mobilization to polysomes might be involved in regulating GP82 expression, as was reported for another *T. cruzi* gene [61]. 

There are at least three known factors that modulate mRNA steady-state level: *cis*-acting elements, *trans*-acting factors, and the apparatus involved in mRNA turnover and degradation [62]. *Cis*-acting elements are non-coding sequences that act from inside the same molecule (intramolecular action). *Trans*-acting factors are diffuse molecules, usually proteins, that act from a different molecule to regulate a target mRNA (intermolecular action) [63]. The fate of transcripts is determined by the interaction of *cis*-acting sequences present in the 3′UTR with specific *trans*-acting protein factors containing RNA-binding domains that subsequently recruit the protein machinery to destroy or stabilize mRNAs [64]. The involvement of GP82 3′UTR in mRNA stability was analyzed using a reporter green fluorescent protein (GFP) fused upstream to the GP82 3′UTR. Parasites transfected with an episomal plasmid carrying this construct had their GFP protein and mRNA levels analyzed, revealing that the 3′UTR was able to downregulate GFP in epimastigotes and upregulate it in metacyclic forms [65]. Similar mechanisms for controlling mRNA stability by 3′UTR sequences have also been described for other TS family members, such as the flagellum-associated surface protein FL-160 (TcS group III) [66], two genes coding for active trans-sialidase enzymes from TcS group I, described by Jager et al., 2008, [67], and another TS member [64]. There are pieces of evidence that stem-loop secondary structures formed in the 3′UTR might be responsible for the interaction with RNA-binding proteins [68]. Prediction of GP82 3′UTR secondary structure was performed *in silico* using mfold program [69], revealing the presence of stem-loop structures; however, the role of these structures was not analyzed so far.

**Figure 3:**
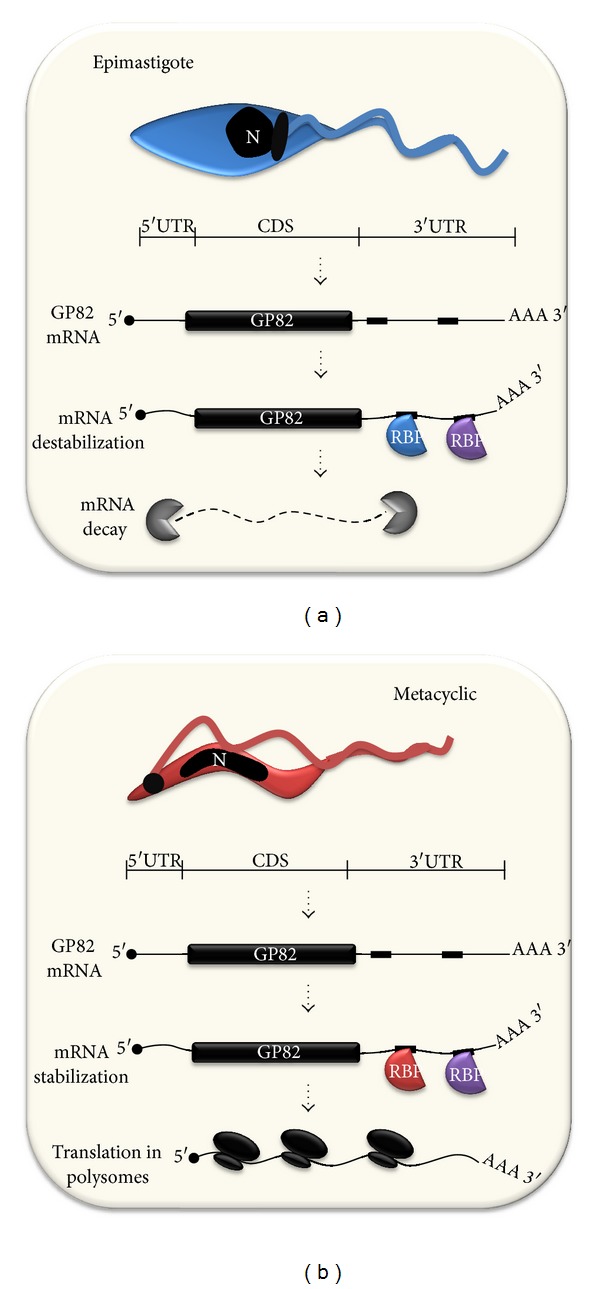
Comparison of GP82 mRNA post-transcriptional control mechanisms in (a) epimastigotes and (b) metacyclic trypomastigotes. In epimastigotes, GP82 mRNA interacts with possibly more than one RNA-binding protein (RBP), which binds to different *cis*-elements in the 3′UTR region (small black rectangles), leading to mRNA destabilization and decay. Conversely, in the metacyclic trypomastigote stage, a different set of RBPs interacts with the *cis*-elements present in the 3′UTR, promoting mRNA stabilization and translation in polysomes.

Regulatory *cis*-acting elements of variable sizes were identified in the 3′UTR of some trypanosomatid genes (reviewed in [62, 70]). In the case of GP82, four step-wise deletions were performed to search for regulatory elements in its 3′UTR. Results indicated that more than one region was responsible for changing GFP mRNA and protein levels in epimastigotes and metacyclic forms [65], suggesting that multiple *cis*-acting elements are present in GP82 3′UTR and might bind to distinct RNA-binding proteins (RBP). The first *trans*-acting factor identified in *T. cruzi *was TcUBP1 (*T. cruzi *uridine binding protein 1), which binds to AU-rich elements of the TcSMUG mRNA leading to its destabilization [71]. In addition to TcSMUG, other 39 transcripts were found bound to TcUBP1 by co-immunoprecipitation assays [68]. One common *cis*-acting element was identified in the 3′UTRs of the majority of these TcUBP1 target mRNAs. This *cis*-element was used to predict novel UBP1 target mRNAs and GP82 was one of them [68]. Therefore, TcUBP1 could be one of the *trans*-acting factors involved in GP82 mRNA stability. A schematic representation of the mechanism controlling *GP82* gene expression is shown in Figure 3.

There are growing pieces of evidence suggesting the presence of post-transcriptional operons in trypanosomes, mediated by the coordinated interaction between *cis*-elements and *trans*-acting factors [68, 72]. It was demonstrated that a group of *T. brucei *stage-regulated proteins share a specific sequence motif in the 3′UTR (reviewed in [72]). Also, two RBPs from *T. cruzi*, TcUBP1, and TcUBP3, preferentially associate with a set of functionally related transcripts bearing the same RNA motif that is recognized by each protein [68]. These post-transcriptional operons could explain how coordinately expression regulation is achieved in organisms where gene-specific transcriptional control is absent.

## 6. Concluding Remarks and Perspectives

GP82 shows a modular organization, with some variation of N-terminal region flanking a conserved central core where the binding sites to mammalian cell and gastric mucin are located. The function of GP82 as adhesin in host cell invasion process could expose the protein to an intense conservative and selective pressure. The potential variability of *GP82* genes suggests that they are not susceptible to mutation. The many isoforms of GP82 and its multiple N-terminal variants suggest that some GP82 family members might display different cellular localizations and functions. The challenge is to ascertain the relationships between *GP82* gene sequences, protein isoforms, and its distinct or overlapping functions.

GP82 is a GPI-anchored surface protein, synthesized as a 70 kDa precursor devoid of *N*-linked sugars and when mature has an apparent molecular weight of 82 kDa. GPI-minus variants accumulate in the ER indicating that GPI anchor acts as a forward transport signal for progressing along the secretory pathway as suggested for *T. cruzi* mucins [73]. Heterologous expression of GP82 into mammalian cells indicated that the requirements for GPI-anchoring are different between *T. cruzi* and mammalian cells. These differences could be targets for the development of parasite-specific therapeutic agents.

Several studies demonstrated that the transcription of GP82 is constitutive and may be regulated at post-transcriptional level, for instance, at translational level and/or mRNA stabilization. GP82 mRNAs are mobilized to polysomes and consequently translated, but only in metacyclic trypomastigotes. It has been suggested that the stabilizing mechanism acting in metacyclic trypomastigotes and the destabilizing mechanism in epimastigotes could be mediated by a cis-acting element present in the 3′UTR of transcripts. A series of step-wise deletions in the 3′UTR was created and results suggest that the mechanism regulating GP82 expression involves multiple elements in the 3′UTR. Interestingly, the 3′UTR of GP82 transcript promotes higher expression of the green fluorescent protein (GFP) reporter in metacyclic trypomastigotes than in epimastigotes. 

In conclusion, while our knowledge of the structure and function of GP82 is large, there still remain many questions to be answered. Additional studies are carried out to analyze the expression, localization, and involvement in host cell invasion of each GP82 variant identified to date.
